# Supplementary material for: Robust Trajectory Generation for Robotic Control on the Neuromorphic Research Chip Loihi
Source: Front Neurorobot. 2020 Nov 26;14:589532. doi: 10.3389/fnbot.2020.589532 (PMC7726255; doi:10.3389/fnbot.2020.589532)
Supplement: Supplementary file 1 [file Data_Sheet_1.PDF]

frontiers

Supplementary Material

1 SUPPLEMENTARY TABLES AND FIGURES

1.1 Figures

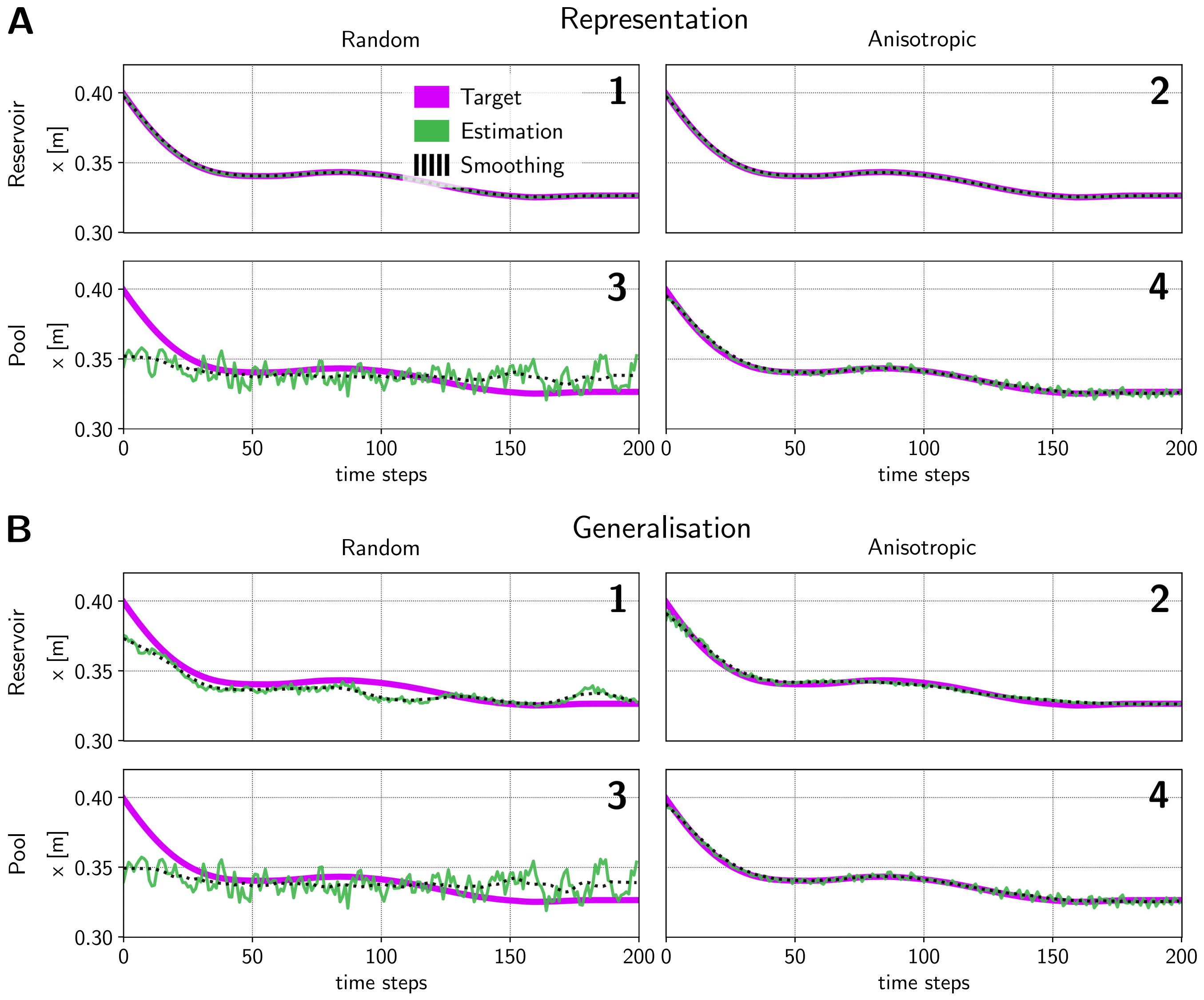

Figure S1. A single trajectory estimation for the x-dimension for all different tasks (representation & generalisation), networks (randomly connected network & anisotropic network) and estimation methods (excitatory reservoir neurons with elastic net regularization & pooling layer neurons).

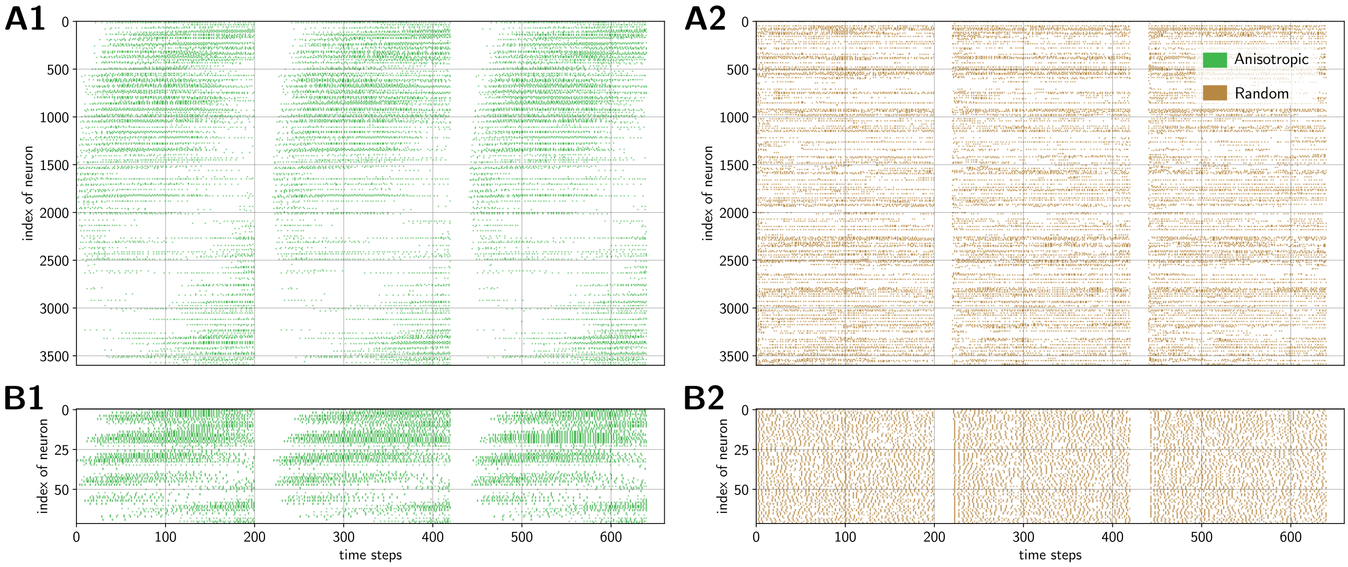

Figure S2. Spike trains of the excitatory reservoir neurons **A** compared with the spike trains of the pooling layer neurons **B**. In the anisotropic network (green) the stream-like structure of the excitatory reservoir neurons are reflected in the pooling layer.

1.2 Tables

Parameter		NEST	Loihi
temporal resolution	dt	$0.1\,ms$	N/A
excitatory neurons	$npop_E$	3600	3600
inhibitory neurons	$npop_I$	900	900
membrane capacitance	C_m	$250.0\,pF$	N/A
leak conductance	g_L	$25.0\,nS$	N/A
threshold potential	v_{th}	$-55.0\,mV$	64000
resting potential	E_L	$-70.0\,mV$	0
reset potential	v_{reset}	$-70.0\,mV$	0
refractory period	t_{ref}	$2.0\,ms$	2
synaptic time constant (exc.)	τ_{exc}	$5.0\,ms$	N/A
synaptic time constant (inh.)	τ_{inh}	$5.0\,ms$	N/A
current decay	τ_I	N/A	380
voltage decay	τ_v	N/A	400
synaptic delay	d	$1.0\,ms$	1
synaptic weights (excitatory)	J^{exc}	$40\,pA$	12
synaptic weights (inhibitory)	J^{inh}	$-160\,pA$	48
connection probability	p_{conn}	0.05	0.05
perlin scale	κ_{perlin}	4	4
gaussian sigma (exc.)	σ_E	12	12
gaussian sigma (inh.)	σ_I	9	9
shift magnitude	n_{shift}	1	1

Table S1. Comparison of parameters used for the `NEST` and the Loihi simulation. Both implementations use leaky integrate-and-fire neurons with current-based synapses. The `NEST` model has an additional alpha-function shaped synaptic current rise, which is not available on Loihi.
